# Innovative Approaches to EMT-Related Biomarker Identification in Breast Cancer: Multi-Omics and Machine Learning Methods

**DOI:** 10.3390/biotech14030075

**Published:** 2025-09-22

**Authors:** Ghazaleh Khalili-Tanha, Alireza Shoari

**Affiliations:** 1 Department of Medical Genetics and Molecular Medicine, School of Medicine, Mashhad University of Medical Sciences, Mashhad 91388-13944, Iran; 2 Department of Cancer Biology, Mayo Clinic Comprehensive Cancer Center, Jacksonville, FL 32224, USA

**Keywords:** breast cancer, epithelial–mesenchymal transition, multi-omics data, artificial intelligence, biomarkers

## Abstract

Breast cancer is the most prevalent cancer among women and is challenging to diagnose and treat due to its diverse subtypes and stages. Precision medicine aims to improve early detection, prognosis, and treatment planning by identifying new clinical biomarkers. The review emphasizes the importance of using cutting-edge technology and artificial intelligence (AI) to identify new biomarkers associated with epithelial–mesenchymal transition (EMT). During EMT, epithelial cells transform into a mesenchymal state, a process driven by genetic and epigenetic alterations that facilitate cancer progression. The review discusses how statistical analysis and machine learning methods applied to multi-omics data facilitate the discovery of novel EMT-related biomarkers, thereby advancing therapeutic strategies. This conclusion is supported by numerous clinical and preclinical studies on breast cancer.

## 1. Introduction

Breast cancer is one of the most common cancers affecting women worldwide [1]. It arises from the breast tissue, typically from the lining of the milk ducts or lobules, and while it predominantly affects women, men can also develop breast cancer [2]. The incidence rate of breast cancer varies globally but has been increasing over the years due to factors such as lifestyle changes, environmental influences, and improved detection methods [3]. In the United States, for instance, the American Cancer Society estimated about 310,720 new cases of invasive breast cancer in women in 2024 [4]. Breast cancer mortality has been declining in many developed countries, largely due to early detection and improved treatment methods; despite this, it remains the second leading cause of cancer death among women [5].

Breast cancer can be classified into several types, including (i) ductal carcinoma in situ (DCIS) which is non-invasive cancer where abnormal cells are found in the lining of a breast duct but have not spread outside the duct; (ii) invasive ductal carcinoma (IDC) which is the most common type, where cancer cells spread beyond the ducts into other parts of the breast tissue; (iii) invasive lobular carcinoma (ILC) that cancer cells spread from the lobules to surrounding breast tissues [6]; (iv) triple-negative breast cancer (TNBC) that lacks estrogen, progesterone, and HER2 receptors, making it harder to treat [7]; (v) HER2-positive breast cancer which characterized by overexpression of the HER2 protein, that promotes cancer cell growth [8].

Clinical biomarkers are used to help diagnose, predict, and monitor the treatment response in breast cancer patients. Estrogen receptor (ER) and progesterone receptor (PR) positivity indicate that the cancer cells may receive signals from these hormones, promoting their growth [9]. Overexpression of the HER2 protein can lead to more aggressive cancer, and HER2-positive cancers may benefit from targeted therapies like trastuzumab (Herceptin) [10]. Higher levels of *Ki-67* gene indicate a higher growth rate of the cancer cells [11]. Genetic mutations in these *BRCA1* and *BRCA2* significantly increase the risk of developing breast cancer [12]. Expression of PD-L1 can be a biomarker for response to immunotherapy in certain breast cancer subtypes [13,14].

Epithelial–mesenchymal transition (EMT) is a cellular program in which epithelial cells lose polarity and adhesion properties, acquiring mesenchymal traits that enhance motility. While essential in development and wound repair, EMT dysregulation contributes to fibrosis and, importantly, cancer progression. In breast cancer, type-3 EMT (Type 3 EMT refers to oncogenic EMT in carcinoma cells, distinct from Type 1 developmental EMT and Type 2 wound-healing/fibrotic EMT) arises from genetic/epigenetic alterations and tumor microenvironmental cues—including hypoxia, growth factors, and inflammatory cytokines—that collectively drive invasion and metastasis [15,16].

Here, modern methods for the detection of EMT biomarkers in breast cancer are going to be discussed. To offer a thorough grasp of EMT processes, it explores the integration of multi-omics technologies, including genomics, transcriptomics, proteomics, and metabolomics. The review also highlights the application of advanced machine learning algorithms to analyze complex datasets, identify novel biomarkers, and predict disease progression. By combining these innovative approaches, the article aims to offer insights into more accurate diagnostic tools and targeted therapies for breast cancer.

Breast cancer is a highly heterogeneous disease encompassing distinct molecular subtypes—luminal A, luminal B, HER2-enriched, triple-negative, and basal-like—each defined by unique gene-expression profiles and showing different therapeutic responses and prognoses [17]. Recognizing these subtypes is critical because they influence treatment selection, resistance mechanisms, and the relevance of EMT-associated biomarkers discussed in this review. To maintain focus on these clinically meaningful categories, we streamlined demographic statistics and retained only those essential for understanding molecular heterogeneity and its impact on EMT-related multi-omics analyses.

## 2. Epithelial–Mesenchymal Transition (EMT)

EMT is a biological process where epithelial cells undergo significant morphological changes, adopting a mesenchymal phenotype. This transformation involves a shift from a highly organized, polarized, and adhesive cell structure to a more motile and invasive phenotype [18]. EMT is crucial for various physiological processes, including embryogenesis, wound healing, and tissue regeneration; however, it also plays a significant role in pathological conditions, most notably cancer progression [19]. Characterized by the downregulation of epithelial markers (such as E-cadherin) and the upregulation of mesenchymal markers (such as N-cadherin and vimentin), EMT involves extensive changes in cell morphology, signaling pathways, and gene expression profiles [20]. EMT is driven by several transcription factors, including Snail, Slug, Twist, and Zeb1/2, which orchestrate the reprogramming of the epithelial phenotype [21]. Key characteristics of EMT are loss of epithelial markers, where epithelial cells lose cell–cell adhesion molecules, such as E-cadherin, and on the other hand, cells gain mesenchymal markers like N-cadherin and vimentin; Actin cytoskeleton is reorganized, contributing to changes in cell shape and increased motility, and cells acquire the ability to invade extracellular matrices and migrate [22].

EMT is implicated in several stages of cancer development and progression. Disruption of epithelial cell junctions and loss of polarity can lead to increased cellular proliferation and survival, contributing to tumor initiation [23]. EMT endows cancer cells with migratory and invasive capabilities, enabling them to breach the basement membrane and invade surrounding tissues and facilitating the entry of cancer cells into the bloodstream or lymphatic system, aiding in the dissemination to distant organs [24]. EMT-induced cells often exhibit resistance to apoptosis, allowing them to survive in the hostile microenvironment of distant metastatic sites; additionally, EMT is associated with increased resistance to conventional chemotherapy and targeted therapies, complicating treatment strategies [25]. Key transcription factors such as Snail, Slug, Twist, and Zeb are upregulated during EMT, driving the repression of epithelial markers and the induction of mesenchymal traits, and various signaling pathways, including TGF-β, Wnt, Notch, and Hedgehog, play critical roles in regulating EMT [26,27].

Proteases drive EMT by degrading the extracellular matrix, activating signaling pathways, cleaving cell-adhesion molecules, and altering cell-surface receptors to enhance cellular plasticity [28,29]. Matrix metalloproteinases (MMPs) degrade extracellular matrix components, a process vital for normal tissue remodeling but also a key driver of cancer metastasis [30]. MMPs also play a significant role in pathological processes, such as cancer metastasis, and one of the key processes in cancer metastasis is the EMT, where epithelial cells acquire mesenchymal properties, enhancing their migratory and invasive abilities [31]. Specific MMPs are involved in regulating EMT through various mechanisms, and MMPs degrade various components of the ECM, creating pathways for cancer cells to invade surrounding tissues and disseminate to distant sites [32]. MMPs can release bioactive growth factors sequestered in the ECM, such as TGF-β, which further promote EMT and cancer progression [33]. MMP-2 degrades type IV collagen, a major component of basement membranes, facilitating cell invasion and migration, and its activity is often upregulated during EMT, promoting the breakdown of ECM and enabling tumor cells to penetrate surrounding tissues [34]. Similarly to MMP-2, MMP-9 degrades type IV collagen and is involved in ECM remodeling, and MMP-9 activity is linked to increased cell motility and invasion during EMT [35]. MMP-3 can degrade various ECM components and activate other MMPs, such as MMP-9, and it is implicated in promoting EMT by inducing the expression of mesenchymal markers (e.g., vimentin) and repressing epithelial markers (e.g., E-cadherin) [36]. Furthermore, MMP-7 is involved in the degradation of ECM components and the release of growth factors that can promote EMT, and it plays a role in the cleavage of E-cadherin, leading to the disruption of cell–cell adhesion and enhanced cell motility [37]. Moreover, MMP-14 is a membrane-type MMP that activates pro-MMP-2 and degrades ECM components directly, and it is critical for cell migration and invasion during EMT [38]. These mechanistic insights align with multi-omics and machine-learning studies that highlight MMP-related EMT signatures in breast cancer. For example, XGBoost models have identified MMP3, MMP9, and MT1-MMP (MMP14) transcripts and proteomic features as predictors of invasion and poor prognosis.

**Figure 1:**
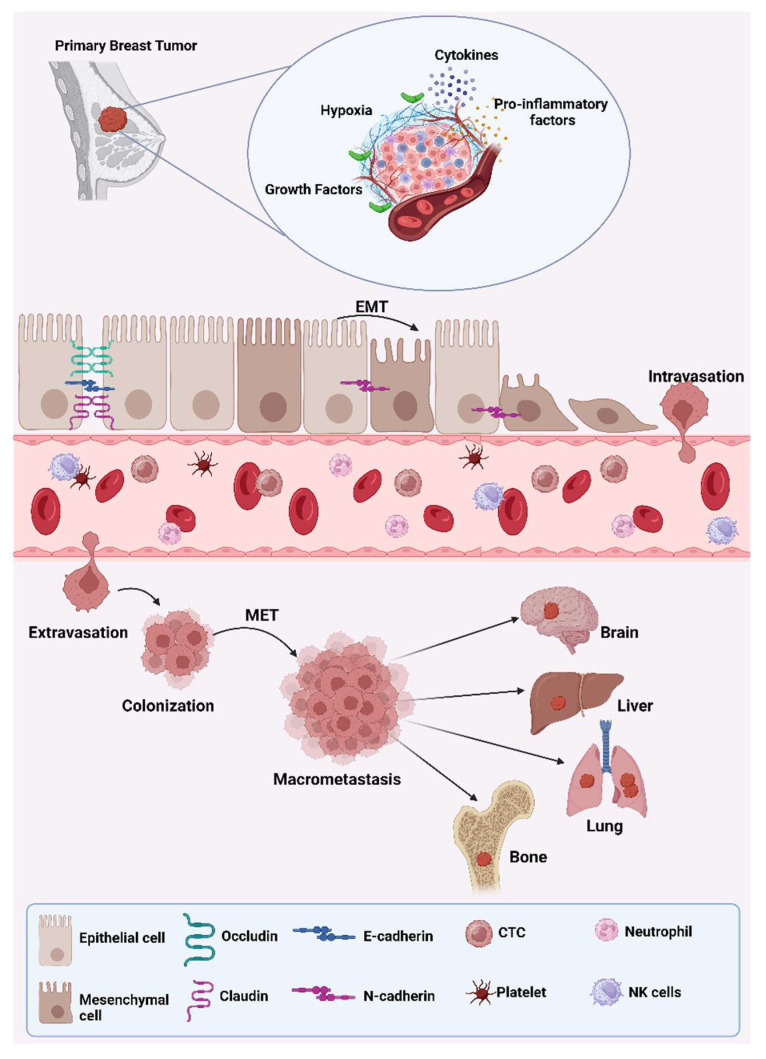
The EMT-MET model describes the process of cancer metastasis. Epithelial cancer cells undergo epithelial–mesenchymal transition (EMT), losing cell–cell junctions and gaining invasive abilities. These cells then enter the bloodstream (intravasation) and must survive circulation to reach a target organ. Upon arrival, they exit the bloodstream (extravasation) and invade the tissue. For these cells to form detectable and potentially dangerous macro-metastases, they must undergo mesenchymal–epithelial transition (MET). (illustration created with BioRender.com) (https://app.biorender.com, accessed on 13 August 2025).

Understanding the mechanisms of EMT has significant therapeutic implications, such as developing inhibitors that target key EMT transcription factors or signaling pathways to prevent or reverse EMT in cancer cells and strategies to overcome EMT-associated drug resistance by combining EMT inhibitors with conventional therapies [21]. EMT markers can serve as potential biomarkers for early detection of metastasis and prognosis prediction in breast cancer patients [39]. EMT is a critical biological process that occurs in both normal physiological and pathological situations. In the context of cancer, EMT plays a pivotal role in tumor initiation, invasion, metastasis, and resistance to therapy. Targeting EMT and its regulatory mechanisms holds promise for improving cancer treatment and patient outcomes (Figure 1).

## 3. Biomarkers and Analyzing Multi-Omics Data

Identifying novel biomarkers, particularly those linked to EMT in breast cancer, is crucial for improving diagnosis, prognosis, and treatment personalization [40]. Prognostic biomarkers help stratify patients based on risk profiles and guide more tailored therapies [41]. In breast cancer, EMT-associated biomarkers are especially valuable because they capture tumor aggressiveness, metastatic potential, and therapy resistance [42].

Multi-omics approaches provide a more complete view of EMT regulation in breast cancer. Genomic data reveal mutations and alterations associated with EMT [43], while transcriptomic profiles capture the expression shifts in EMT-related genes [44]. Proteomic analysis identifies changes in protein abundance and signaling cascades driving EMT [45], and metabolomics reflects metabolic reprogramming that accompanies the transition to a mesenchymal state [46].

Integrating these data layers enables the identification of robust EMT biomarkers and regulatory networks underlying breast cancer progression [39]. Compared with generic big data approaches, omics-driven strategies in breast cancer highlight clinically relevant EMT biomarkers. For instance, multi-omics integration has revealed EMT signatures linked to poor survival and resistance to chemotherapy, offering targets for prognostic tools and therapeutic intervention [47].

Traditional statistical methods such as *t*-tests, ANOVA, and regression analysis remain useful for analyzing omics data [48], and dimensionality reduction approaches like PCA help identify patterns but may oversimplify complex EMT-related biology [49]. Hierarchical clustering can group EMT gene or protein signatures [50], while pathway enrichment methods such as GSEA and KEGG highlight dysregulated EMT pathways [51]. However, these approaches often struggle with the high dimensionality and cross-omics integration challenges of EMT datasets [52].

Machine learning methods are increasingly applied to overcome these challenges in breast cancer EMT research. Supervised learning (e.g., support vector machines, random forests) and unsupervised clustering approaches identify EMT-related signatures from high-dimensional omics data [53]. Deep learning architectures, such as convolutional neural networks (CNN), extract multi-level EMT features [54], and AI-based integration frameworks like MOFA enable cross-omics analysis of EMT regulation [55]. Natural language processing (NLP) further accelerates biomarker discovery by mining EMT-related associations from literature [56].

AI methods not only enhance EMT biomarker discovery but also improve prediction of clinical outcomes in breast cancer [57]. By capturing non-linear, multi-layered interactions across omics levels, AI models provide a scalable and powerful framework for identifying EMT-related diagnostic, prognostic, and therapeutic biomarkers.

## 4. Integrative Multi-Omics Analysis with Artificial Intelligence

**Figure 2:**
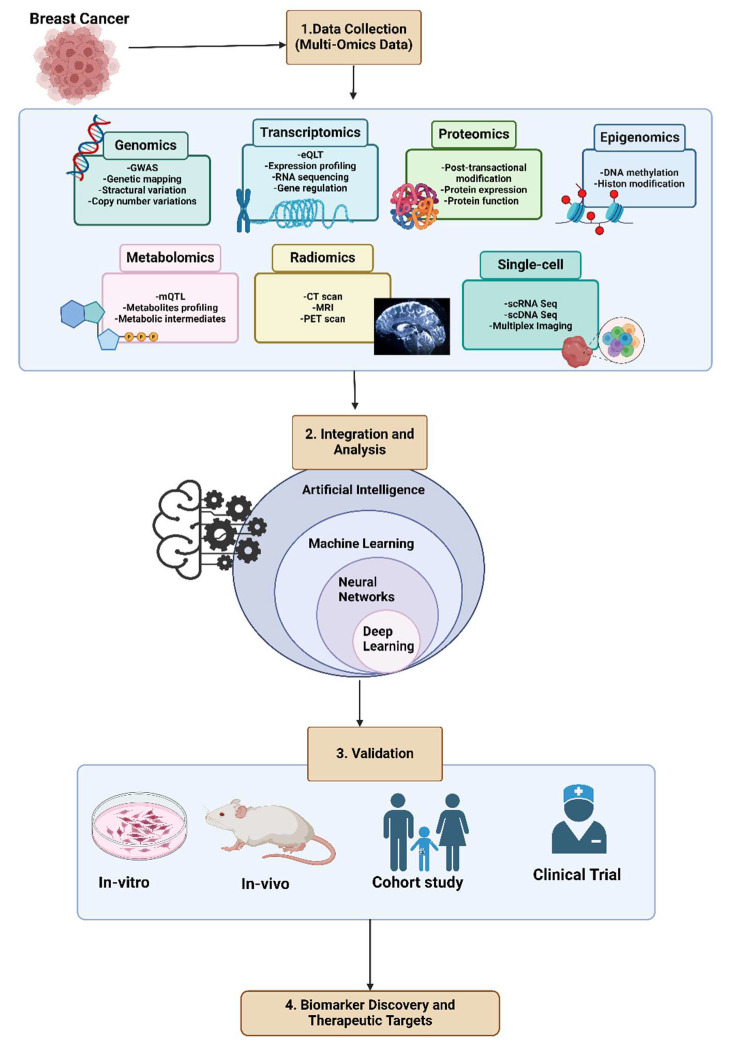
The identification of EMT-related biomarkers involves analyzing extensive breast cancer data through an integrated multi-omics approach. This includes genetics, epigenetics, transcriptomics, proteomics, metabolomics, single-cell multi-omics, and radiomics, analyzed using statistical and machine learning techniques. The findings from this comprehensive analysis need validation through further experiments, such as in vitro, in vivo, cohort studies, and clinical trials. These studies aim to identify potential prognostic, diagnostic, and predictive biomarkers and to develop therapeutic strategies for breast cancer. (illustration created with BioRender.com) (https://app.biorender.com, accessed on 13 August 2025).

Finding novel biomarkers in cancer is critical for early diagnosis, prognosis, and treatment personalization. The integration of big data and artificial intelligence (AI) significantly enhances this process and develops personalized treatment plans based on individual biomarker profiles. This means treatments can be tailored to the specific genetic and molecular makeup of a patient’s cancer, potentially improving efficacy and reducing side effects. As shown in Figure 2, the process involves five steps of AI application in analyzing biological data.

Data Collection: This initial step involves gathering multi-omics data, such as genomics, epigenomics, transcriptomics, metabolomics, proteomics, single-cell multi-omics, spatial transcriptomics, etc., from various biological databases [58]. Analyzing each type of omics data provides valuable insights into the biological and molecular pathways involved in disease progression. Several databases provide this biological data, with Gene Expression Omnibus (GEO) and The Cancer Genome Atlas (TCGA) being the most commonly used. These databases offer comprehensive genomic, transcriptomic, epigenomic, and proteomic data across different types of cancer [59,60]. Additionally, some databases focus on specific cancers, such as Breast Cancer Gene Expression Miner (bc-GenExMiner), which specifically provides gene expression data related to breast cancer obtained through RNA sequencing methods and DNA microarrays [61]. METABRIC, the Molecular Taxonomy of Breast Cancer International Consortium, is a comprehensive initiative focused on understanding breast cancer at the molecular level. This extensive study provides detailed genomic, transcriptomic, and clinical data from a large cohort of breast cancer patients. The project was generously funded by Cancer Research UK, the Canadian Breast Cancer Foundation BC/Yukon, and the British Columbia Cancer Foundation [62].

Pre-processing: It is an essential step for raw data and includes data filtering, batch effect removal, systematic normalization, and quality checks. These steps are crucial because they significantly influence the outcomes of integrative analyses. In particular, data filtering is vital for reducing noise and minimizing features, which is important given the computational demands of most integrative methods [63]. LASSO, Ridge regression, and Elastic Net regularization are advanced pre-processing techniques used to enhance machine learning models by selecting relevant features. The pre-processing of various types of multi-omics data requires specialized tools and packages [64].

The third step involves feature selection and feature extraction, which are methods used to decrease the number of features in a dataset. Feature selection involves picking a subset of the original features, removing attributes or features that introduce noise, irrelevance, or redundancy, thereby improving the accuracy of data sample classification. While feature extraction changes the original features into a new set [65,66]. Common techniques include Independent Component Analysis (ICA) and Principal Component Analysis (PCA). The benefits of both techniques include simplifying and enhancing the model’s effectiveness, improving interpretability, reducing dimensionality, and resulting in a more concise and cohesive statement [67,68].

Model Training: The process of model training leverages both supervised and unsupervised learning techniques to teach the AI system [69]. Supervised learning uses labeled data to help the AI learn the relationships between input features (biomarkers) and output labels (conditions or outcomes). Common supervised ML algorithms for classification include Naïve Bayes (NB), Random Forest (RF), Support Vector Machines (SVM), and K-Nearest Neighbors algorithm (k-NN) [70,71,72]. Unsupervised learning, on the other hand, helps the AI find hidden patterns or intrinsic structures in the data without explicit labels. By applying these methods, the AI becomes capable of recognizing and predicting the connections between biomarkers and various medical conditions. Unsupervised learning methods like clustering and dimensionality reduction are frequently used in biomarker discovery [73,74].

Validation and evaluation: They are crucial stages in analyzing AI models, ensuring their performance and generalizability. Validation assesses a trained model’s ability to handle new, unseen data, enhancing its capacity to generalize effectively. This process involves evaluating metrics such as accuracy, sensitivity, and specificity using data separate from the training set. There are two main types of validation: internal and external. Internal validation, typically performed through cross-validation, involves dividing the data into training and test sets to evaluate the accuracy of the model. External validations, such as in vivo and in vitro experiments, along with in-house cohort studies [75,76]. Successful cross-layer modeling requires careful technical control [77]. Batch effects arising from different sequencing platforms or acquisition dates should be mitigated using tools such as ComBat or Harmony, and missing-layer samples can be handled through imputation methods or model architectures that tolerate incomplete views (matrix factorization, variational autoencoders). Multi-view learning frameworks—including early-fusion approaches that merge features before modeling and late-fusion methods such as MOFA that learn latent factors across omics layers—should be selected according to study goals and sample size. To avoid model overfitting and information leakage, preprocessing and feature selection must be performed inside the training folds during cross-validation, and feature-stability analyses (e.g., bootstrapping or repeated cross-validation) are essential to confirm robustness of selected biomarkers. These practices help ensure that predictive EMT signatures derived from integrated datasets remain reproducible and clinically meaningful.

## 5. Machine Learning: Revolutionizing Multiomics Data Interpretation

Recent advances in analytical technologies have generated an exponential increase in data from diverse omics platforms. Statistical methods and AI, particularly machine learning (ML) and deep learning (DL) algorithms, have proven highly effective in analyzing these large-scale biological and clinical datasets. By uncovering complex patterns, they have facilitated the identification and validation of EMT-related biomarkers that hold promise for personalized treatment decisions and the development of targeted therapies. In this review, we summarized and compiled novel biomarkers identified through multi-omics integration using ML approaches (Table 1). Relevant studies were identified from PubMed, Web of Science, and Scopus using combinations of “breast cancer,” “epithelial–mesenchymal transition,” “multi-omics,” “biomarkers,” and “machine learning.” Given the heterogeneity of endpoints, algorithms, and datasets, strict inclusion/exclusion criteria were not applied as in a systematic review; rather, studies were selected to illustrate the diversity of approaches and key findings in this emerging field.

While multi-omics and ML approaches have uncovered promising EMT-related biomarkers, their clinical utility ultimately depends on translation into deployable assays. Several avenues are currently being explored. Immunohistochemistry (IHC) panels incorporating recurrent EMT markers such as E-cadherin, N-cadherin, vimentin, and fibronectin offer standardized diagnostic and prognostic assessment, provided staining reproducibility and concordance with molecular EMT signatures are demonstrated [78,79]. Although the trials and multi-omics analyses summarized in Table 1 provide important leads, their biomarker findings vary in sample size, cohort diversity, and analytical rigor. Many rely on retrospective datasets or single-center cohorts, which can limit reproducibility. Independent external validation is often incomplete, and assay platforms differ in sensitivity and specificity across studies. These factors highlight the need for larger prospective trials with standardized protocols, cross-platform benchmarking, and transparent data sharing to confirm the clinical utility of EMT-related biomarkers.

qPCR-based gene signatures, such as those derived from miR-21, miR-222-3p [80], and ESRP1/2, represent rapid and low-cost clinical tests, though they require rigorous cross-cohort validation and benchmarking against existing prognostic models [81,82]. Extracellular vesicle (EV)-based proteomics has shown that proteins such as FAK, MEK1, and fibronectin enriched in circulating EVs can serve as minimally invasive EMT biomarkers, but clinical translation necessitates robust EV isolation protocols, reproducible proteomic quantification, and adherence to laboratory standards [82]. Similarly, radiomics and imaging pipelines applying ML to MRI-based radiomic features have successfully predicted EMT status and correlated it with outcomes; however, their clinical adoption will require harmonization of imaging protocols, standardized feature extraction, and integration into hospital PACS systems [83].

Recent studies have leveraged machine learning (ML) and multi-omics data to identify epithelial–mesenchymal transition (EMT)-related biomarkers in breast cancer, particularly in aggressive subtypes such as triple-negative breast cancer (TNBC). Thalor et al. analyzed GEO gene expression datasets using multiple ML algorithms, including SVM, kNN, RF, DT, LR, and XGBoost. XGBoost achieved the highest accuracy and AUC with the top 25 features (CX-25), including BCHE, ATP7B, PPP4R4, TFF1, PTGFR, TTYH1, and SERPINA6, while the driver dataset (DX-20) highlighted CDKN2A, WIF1, ZNF521, MUC16, WNK4, COL2A1, and S100A7. Kaplan–Meier analysis identified S100B and POU2AF1 as potential EMT-related prognostic genes, linked to metastasis-associated pathways such as PI3K-AKT, Wnt, MAPK, and TGF-β. This study underscores the power of ML to uncover biomarkers that survive external validation and are directly connected to EMT processes [84]. Building on this concept, Rozova et al. explored how the microenvironment influences EMT dynamics. Using ML to analyze mesenchymal breast carcinoma cells on substrates with varying stiffness, they observed EMT to MET mediated by E-cadherin localization and vimentin expression, demonstrating the critical role of biomechanical cues in regulating EMT markers. These findings complement gene-based analyses, emphasizing that EMT is not only genetically regulated but also microenvironment-dependent [78]. Further integrating molecular layers, Villemin et al. identified an EMT-related splicing signature in basal-like TNBC. ML analysis revealed that splicing regulators RBM47 and ESRP1/2 control EMT-associated alternatively spliced variants. Low RBM47 expression correlated with poor prognosis, highlighting its potential as a clinically relevant EMT biomarker [85]. Similarly, XGBoost-based models on TCGA datasets identified metastasis marker genes (RGS7, SPPL2C, KRT23) that would have been missed by traditional statistics, illustrating how ML captures non-linear interactions and complex feature relationships relevant to EMT [86].

Integration of EMT markers with immune profiling was demonstrated by Chen et al., who applied unsupervised clustering to TNBC datasets (TCGA, GEO) to define immune subtypes. Correlation with EMT markers (CDH2, FN1, CDH1, VIM) revealed higher EMT activity in subtypes with poor prognosis. Random forest models further predicted clinical outcomes and potential immunotherapy response, highlighting the translational value of integrating EMT biomarkers with ML-based multi-omics analysis [79].

Circulating biomarkers also provide insight into EMT regulation. Triantafyllou et al. identified miR-21 as a common EMT-related molecule across breast cancer subtypes. LZTFL1, a target of miR-21, regulates EMT via E-cadherin and actin cytoskeleton interactions, illustrating how post-transcriptional mechanisms can serve as prognostic EMT biomarkers [81,87]. In parallel, Gou et al. used ML to assess tumor microenvironment (TME) profiles in 491 TNBC patients. TME-related gene (TRG) scores correlated with EMT markers and predicted immunotherapy responses, emphasizing the integration of multi-omics and ML for both prognostic and predictive insights [88]. Proteomic analyses further highlight EMT biomarkers. Kothari et al. applied ML algorithms (SCM, DT, RF) to TCGA data, identifying MFGE8 [89] and TBC1D9 as EMT-associated markers that differentiate TNBC from non-TNBC and correlate with prognosis. Similarly, proteomic profiling of circulating sEVs in breast cancer patients revealed upregulated FAK, MEK1, and fibronectin, which could serve as noninvasive diagnostic EMT markers [82].

Hypoxia and metabolism-driven EMT have also been studied using multi-omics integration. Li et al. constructed a hypoxia- and lactate metabolism-related prognostic model (HLMRPM) using TCGA and GEO datasets. Genes such as DARS2, ESRP1, TH, and SLC2A1 modulated EMT pathways, offering predictive insights into survival and therapeutic response [90]. Boolean network models also predicted hybrid EMT cellular phenotypes, further illustrating the utility of ML in classifying complex EMT states [91].

Finally, deep learning and imaging-based approaches have enabled single-cell quantification of EMT phenotypes. Malik et al. applied deep neural networks integrating multi-omics and clinical features to stratify patients into risk groups and predict drug response. Key EMT-related genes (CDH1, PIK3CA, TP53, EFHD1) were highlighted, demonstrating the value of ML in connecting molecular EMT signatures to clinical decision-making [92].

**Table 1:** Potential EMT-related biomarkers in breast cancer identified through machine learning-based integrated analysis of multi-omics data.

Biomarker(s)	Role in Breast Cancer	Dataset(s)	ML Method(s)	Validation	Cohort Size	Clinical Context (Assay)	Outcome	Ref.
BCHE, ATP7B, PPP4R4, TFF1, PTGFR, TTYH1, SERPINA6, CDKN2A, WIF1, ZNF521, MUC16, WNK4, COL2A1, S100A7, S100B, POU2AF1	Prognostic (multi-gene EMT-associated panel)	GEO	XGBoost	Internal CV; Kaplan–Meier survival analysis	n = 623 TNBC and 527 non-TNBC samples (GEO cohorts)	Prognostic; RNA-seq, microarray	Higher expression is associated with better survival	[84]
E-cadherin (CDH1), Vimentin (VIM)	Prognostic (classical EMT markers)	ECM Select Array	Hierarchical clustering	Experimental (Spearman correlation, *t*-test)	Cell line/tissue assays	Prognostic; IHC, array-based	Worse prognosis due to EMT features	[78]
RBM47, ESRP1/2	Prognostic (RNA splicing regulators of EMT)	GEO	Random Forest, Cox regression	Internal CV; Log-rank, Wilcoxon tests		Prognostic; RNA-seq, microarray	Worse prognosis in basal-like breast cancer	[85]
RGS7, SPPL2C, KRT23	Prognostic (linked to EMT signaling)	TCGA, DisGeNET, KEGG	XGBoost	Internal CV; Kaplan–Meier survival analysis	TCGA: n= 22 samples (metastasis to other organs)	Prognostic; RNA-seq	Worse prognosis	[86]
CDH2, FN1, CDH1, VIM	Prognostic & Predictive (epithelial–mesenchymal switch signature)	TCGA, GEO, METABRIC	Random Forest, Consensus Clustering	Internal CV (TCGA), External validation (METABRIC)	TCGA: n = 116 TNBC; GEO: 815 TNBC METABRIC: n = 313 (ER- and HER2-negative BC)	Prognostic/Predictive; RNA-seq, IHC	Response to immune checkpoint blockade (ICB) and better survival	[79]
miR-21, miR-148b, miR-144, miR-203a, miR-140	Prognostic (EMT-related miRNA)	miRecords, miRTarBase, TarBase	Linear SVM	Internal CV; Fisher’s exact test	n = 66 (primary breast cancer)	Prognostic; qPCR, RNA-seq	miR-21, prognostic marker of worse outcome. miR-148b, miR-144, miR-203a, miR-140, predictive markers for targeted therapy	[81]
Tumor microenvironment-related gene (TRG) score	Prognostic & Predictive (EMT and immune infiltration)	TCGA, GEO, UCSC Xena	LASSO, OCLR, Cox regression	Internal CV; ROC, PCA; External validation in GEO	GEO: multiple cohorts	Prognostic/Predictive; RNA-seq	Low TME-related gene scores are associated with improved prognosis and better response to immunotherapy.	[88]
MFGE8	Diagnostic & Prognostic (linked to EMT signaling)	TCGA, KM Plotter	SVM, Decision Tree, Random Forest	Experimental (qPCR, LC-MS/MS); Internal validation	TCGA: n = 140 TNBC and 737 non-TNBC	Diagnostic/Prognostic; qPCR, proteomics	MFGE8 overexpression is associated with poor prognosis	[89]
Fibronectin, FAK, MEK1	Diagnostic (EMT-related adhesion/migration proteins)	RPPA, immunoblotting, EM	k-NN, Logistic Regression	Experimental (*t*-test, ROC)	Cell lines, patient tissue	Diagnostic; RPPA, IHC	Protein clusters distinguish sample types; some predict relapse and therapy response	[82]
DARS2, SLC2A1, ESRP1, TH, MAFF	Prognostic (EMT-related metabolic & splicing regulators)	TCGA, GEO, UCSC Xena	Cox regression, RSF	Internal CV; ROC, TIDE; External validation GEO	TCGA: n = 1113 (patients with overall survival (OS) time longer than 30 days); GEO: 327	Prognostic; RNA-seq, bioinformatics	worse overall survival in patients with high lactate-hypoxia scores	[90]
GATA3, KRT6, ACTA2, CDH1	Diagnostic & Prognostic (canonical EMT transcription factors)	TCGA, METABRIC	Neural Network (Cox-nnet)	Internal CV; Experimental (IMC imaging)	TCGA: n = 159 (TNBC), n = 599 (Luminal A); METABRIC: n = 299 (TNBC), 1369 (Luminal A)	Diagnostic/Prognostic; RNA-seq, IMC	KRT6 and ACTA2 over-expression and CDH1 under-expression show poor prognosis.	[93]
CDH1, PIK3CA, TP53, EFHD1	Prognostic & Predictive (partly EMT-related)	TCGA, GDSC	Naïve Bayes, SMO, RF, k-NN	Internal CV; STRING/Cytoscape validation	TCGA: n = 1000	Prognostic/Predictive; RNA-seq, microarray	Worse survival and potential treatment response	[92]
miR-222-3p	Diagnostic & Prognostic (EMT-associated miRNA)	TCGA, GEO, miRWalk	OCLR	Internal CV; ROC, Kaplan–Meier	TCGA: n= 1103; GEO: multiple	Diagnostic/Prognostic; qPCR, RNA-seq	Higher miR-222-3p expression indicates worse prognosis.	[80]
Quantitative EMT score (epithelial–mesenchymal traits)	Diagnostic (phenotypic EMT scoring)	DHM imaging	AdaBoost, SVM	Experimental (*t*-test, post hoc analysis)	Cell lines, tissue samples	Diagnostic; digital holographic microscopy		[72]
Immune-radiomic models with EMT signatures	Predictive (therapy response prediction)	MRI-based radiomics	ML algorithms (unspecified)	External validation (MRI cohort)	n = 570 (breast MRI)	Predictive; MRI, radiomics	MRI-based model predicts risk of positive margins in BCS.	[83]

**Abbreviation:** Gene Expression Omnibus (GEO), The Cancer Genome Atlas (TCGA) Extreme Gradient Boosting (XGBoost), Immunohistochemistry (IHC), linear Support Vector Machine (SVM), Quantitative real-time PCR (q-PCR), Liquid Chromatography with tandem mass spectrometry (LC-MS/MS), Random forests (RF), Reverse phase protein array (RPPA), random survival forest (RSF), Triple-negative breast cancer (TNBC), Sequential minimal optimization (SMO), K-Nearest Neighbor’s algorithm (k-NN), Genomics of Drug Sensitivity in Cancer (GDSC).

Complementary studies using single-cell imaging and digital holographic microscopy quantified epithelial–mesenchymal scores via SVM and neural networks, providing sensitive assessment of EMT dynamics in TNBC and luminal subtypes [93]. Ma et al. leveraged MRI-based immune-radiomic models to infer EMT status in tumor margins, linking elevated EMT activity to poor surgical outcomes and highlighting ML’s role in bridging imaging and molecular biomarkers [83].

## 6. EMT-Related Biomarkers: Predictive Indicators and Therapeutic Targets

The primary therapeutic strategies in cancer therapy focus on suppressing EMT processes. This is achieved by targeting EMT transcription factors (EMT-TFs), EMT-related signaling pathways, and EMT-related proteins. By doing so, these strategies aim to reduce the risk of metastasis, thereby improving patient outcomes [94]. Despite advancements in cancer research, significant challenges persist in its treatment. The primary treatments—chemotherapy, radiotherapy, and immunotherapy—often face resistance, making therapy less effective. Addressing this resistance is a key issue in oncology. Over the past decade, there has been substantial advancement in targeted cancer therapies. This includes the development and approval of monoclonal small molecule inhibitors, non-coding RNA molecules, antibodies, and natural products, many of which are currently under clinical investigation. These innovations represent a significant leap forward in the fight against cancer [95]. To develop effective targeted therapies, it is crucial to understand the mechanisms behind therapy resistance, including epithelial–mesenchymal plasticity (EMP) and the tumor microenvironment. By reversing the EMT and maintaining the epithelial traits of cancer cells, their sensitivity to chemotherapy and radiotherapy can be enhanced [96].

Developing new cancer therapies centers on targeting EMT transcription factors (EMT-TFs) and their related signaling pathways to prevent or reverse EMT. This approach aims to inhibit the processes that enable cancer cells to become more invasive and spread to other parts of the body. Akbar et al. conducted a study that combined in silico and in vitro analyses. They used publicly available datasets from cell lines and patient tumors for in silico analyses, while in vitro experiments were conducted on human breast cancer cell lines to assess phenotypic plasticity and drug responsiveness. Their findings indicated that knocking down ZEB1 and SNAI2 genes led to decreased sensitivity to the drug Midostaurin and increased sensitivity to Lapatinib in MDA-MB-157 breast cells. They discovered that the CNCL gene list, which includes genes related to stem-cell and EMT characteristics, is indicative of tumor plasticity and influences cytotoxicity profiles, particularly in response to Lapatinib and Midostaurin in breast cancer. This gene list, while not always accurately forecasting patient outcomes, can help determine which patients are likely to respond well to treatment with Taxane drugs before their main therapy [97]. Imani and colleagues found that by targeting specific EMT-related proteins (Zeb 1, Twist 1, and NOTCH1) with miR-34a and the natural compound thymoquinone, they could halt the EMT process in breast cancer cells. This led to a reduction in both cell invasion and metastasis, highlighting a potential therapeutic approach [98]. Addison et al. investigated the expression of EMT-TF networks across various cancer models and human breast tumors. They used conditional knockdown techniques to demonstrate that ZEB1 and ZEB2 are crucial regulators of metastasis. Their research identified that ZEB1/2, TCF4, SNAI2, and TWIST1/2 are commonly and cooperatively upregulated during forced EMT in normal mammary epithelial cells. This upregulation likely occurs due to the suppression of epithelial-specific miRNAs, such as miR200s/203/205, which normally inhibit multiple EMT-TFs. They also discovered that miR200c can target TCF4, in addition to SNAI2 and ZEB1/2. Remarkably, activating the EMT program in non-transformed epithelial cells bestows stem cell-like properties, characterized by the CD24−/CD44+ phenotype. ZEB1/2 and TWIST1/2 were found to be significantly upregulated in these CD24−/CD44+ mammary stem-like cells, with ZEB1 playing a major role in maintaining their mesenchymal status. Furthermore, using Dox-inducible shRNAs, they showed that depleting ZEB1/2 and suppressing EMT at the early stages of tumor growth can block spontaneous lung metastasis. This suggests that administering anti-EMT drugs during early tumor stages could be an effective strategy for preventing metastasis [99]. In another study, researchers treated breast cancer cells with cisplatin and then measured cell migration and changes in EMT markers using methods such as Western blot, migration assays, and immunofluorescent staining. They analyzed RNA expression changes through RNA-seq and confirmed the binding of activating transcription factor 3 (ATF3) to cytoskeleton-related genes using ChIP-seq. They later utilized a paclitaxel and cisplatin combination in treating xenograft mouse models. This study demonstrated that a lower dose of cisplatin specifically inhibited cancer metastasis without significantly affecting tumor growth. The underlying mechanism was identified as cisplatin disrupting a positive feedback loop between TGFβ and FN1, which is crucial for TGFβ activation. This inhibitory effect was lost when ATF3 was disrupted [100]. Tian et al. performed a study to identify natural inhibitors of breast cancer metastasis; researchers used small interfering RNAs (siRNAs) to transiently knock down 591 ERF-coding genes in luminal breast cancer MCF-7 cells. They discovered that depletion of the gene AF9 significantly promoted MCF-7 cell invasion and migration. A metastasis mouse model further confirmed AF9’s suppressive role in breast cancer metastasis. RNA profiling showed that AF9 target genes are enriched in the EMT pathway. The tandem mass spectrometry revealed that AF9 interacts with Snail, a master regulator of EMT, inhibiting Snail’s transcriptional activity in basal-like breast cancer (BLBC) cells. AF9 reconstitutes an active state on Snail’s promoter, recruiting GCN5 or CBP to derepress target genes. Additionally, miR-5694 targets and degrades AF9 mRNA in BLBC cells, further enhancing cell migration and invasion. Notably, AF9 and miR-5694 expression levels in BLBC clinical samples are inversely correlated. Therefore, miR-5694 mediates the downregulation of AF9, promoting metastasis in BLBC. Restoring the expression of the metastasis suppressor AF9 could be a potential therapeutic strategy against metastatic breast cancer [101]. Another study demonstrated that combining doxorubicin with miR34a both in vitro and in vivo synergistically suppresses the progression of doxorubicin-resistant breast cancer. This combination works by decreasing the expression of Snail, which it achieves by inhibiting various signaling pathways, including RAS/RAF/MEK/ERK and Notch/NF-κB. High Snail expression is known to significantly promote cell migration, invasion, and adhesion, likely through the regulation of E-cadherin and N-cadherin. These findings highlight the importance of miR34a in regulating Snail and suggest that co-administering miR34a with doxorubicin could offer a more effective therapeutic strategy against drug-resistant breast cancer in clinical settings [102].

Extensive evidence indicates that targeting EMT-related proteins and signaling pathways can effectively inhibit cancer progression and enhance the sensitivity of cancer cells to therapy. By disrupting these EMT mechanisms, therapies can become more effective, potentially leading to better patient outcomes. Nelson et al. demonstrated that overexpressing membrane N-cadherin enhances chemo-resistance and invasiveness in TNBC. Using an antibody to inhibit pro-N-cadherin increased chemo-sensitivity in the BT549 and SUM159 cell lines [103]. Senthebane et al. found that elevated levels of collagens, laminins, and fibronectin in tumor tissue play a significant role in cancer progression. They demonstrated that using siRNA to knock down collagen and fibronectin, combined with chemotherapy drugs, increased the sensitivity of esophageal and breast cancer cells to these agents. This approach also reduced colony formation and cancer cell migration, underscoring the potential therapeutic benefits of targeting these extracellular matrix components [104]. Ahmad et al. demonstrated that PGI overexpression enhances NF-κB binding to DNA, resulting in the upregulation of Zeb1 and Zeb2. They found that silencing the PGI pathway reversed the EMT process and reduced the aggressiveness of breast cancer cells. Specifically, knocking down PGI/AMF expression in breast cancer cells led to the upregulation of miR-200s, correlating with the reversal of the EMT phenotype. This was consistent with changes in the expression of epithelial markers (E-cadherin) and mesenchymal markers (ZEB1, vimentin, ZEB2), as well as decreased cell aggressiveness, as evidenced by motility, clonogenic, and invasion assays. These results suggest that miR-200s play a role in PGI/AMF-induced EMT, and therefore, approaches to upregulate miR-200s could represent a novel therapeutic strategy for treating highly invasive breast cancer [105]. Zhu et al. demonstrated that TIMP-1 serves as a prognostic and predictive biomarker in breast cancer. Elevated TIMP-1 levels are linked to poorer overall survival, disease-free survival, and resistance to Paclitaxel [106]. The results of a different study indicated that antisense oligonucleotides decreased miR-221 and miR-222 levels in breast cells, enhancing cellular sensitivity to tamoxifen by increasing TIMP-3 expression [107]. Thakur et al. found that targeting MT1-MMP could improve the effectiveness of chemotherapy and radiotherapy in breast cancer patients, especially those with TNBC. MT1-MMP activates a DNA damage response that affects breast cancer resistance to Doxorubicin in both in vitro studies and an animal model [108]. A pilot investigation on breast cancer patients undergoing traditional and hypofractionated radiation therapy suggested that MMPs (specifically MMP-3 and MMP-9) and TIMP4 could be valuable prognostic and predictive biomarkers. They discovered that MMP-3 levels were associated with tumor size, grade, menopausal status, lymph node involvement, and hormonal receptor status. Additionally, the study revealed a correlation between MMP levels and radiation-induced side effects [109]. Yuan et al. found that breast cancer patients who underwent radiotherapy and chemotherapy post-surgery showed decreased MMP-9 levels and elevated TIMP-1 levels in their serum, which correlated with clinicopathological characteristics [110]. Saxena et al. found that Snail, Foxc2, and Twist increased the expression of multiple ABC transporters in breast cancer cells exposed to Doxorubicin [111]. The metastatic breast cancer model resistant to cyclophosphamide exhibited tolerance to apoptosis, and targeting EMT with miR-200 eradicated chemo-resistance [112].

Breast cancer subtypes differ markedly in their response to immune-checkpoint inhibitors. Triple-negative and some basal-like tumors show the highest—but still modest—objective response rates, whereas hormone-receptor–positive and HER2-enriched cancers generally respond poorly [113]. Resistance mechanisms include low tumor mutational burden, immune-suppressive tumor microenvironments, and up-regulation of alternative checkpoints [114]. Multi-omics and ML studies are beginning to identify predictive biomarkers—such as tumor-infiltrating lymphocyte signatures and composite EMT/immune scores—that correlate with ex vivo drug-sensitivity metrics such as IC_50_ values (the drug concentration that inhibits 50% of a specified cellular response or activity). Incorporating these immune-response data with EMT-focused omics can guide patient stratification and combination-therapy design [115].

## 7. Conclusions

The current review highlights the essential role of advanced technology and AI techniques in identifying novel EMT-related biomarkers for breast cancer. These biomarkers hold strong potential for improving early detection, prognosis, personalized treatment, and the development of targeted therapies. Nevertheless, several challenges remain, particularly the heterogeneity of multi-omics data and the complexity of its integration. To move the field forward, it is crucial to benchmark EMT signatures across large-scale public zdatasets such as TCGA and METABRIC and validate them in independent clinical cohorts to ensure reproducibility and generalizability. Future studies should prioritize multi-omics integration models that demonstrate improved calibration and net clinical benefit compared with single-omics approaches. At the same time, assessing feature stability, interpretability, and incorporating decision-curve analyses will be essential for ensuring the robustness and clinical relevance of ML predictions. Finally, encouraging open science practices, including the sharing of datasets, code, and model architectures, will enhance reproducibility and foster collaborative refinement of EMT-focused ML pipelines. By aligning biomarker discovery with these practical steps, the integration of omics and AI-driven methods can advance from theoretical promise to clinically actionable strategies that improve patient stratification, therapeutic decision-making, and precision oncology.

## Data Availability

No new data were created or analyzed in this study.
